# Non-mucinous adenocarcinomas and squamous cell carcinomas of the anal region masquerading as abscess or fistula: a retrospective analysis and systematic review of literature

**DOI:** 10.1007/s00432-021-03747-8

**Published:** 2021-08-02

**Authors:** Aysun Tekbaş, Henning Mothes, Utz Settmacher, Silke Schuele

**Affiliations:** 1 Department of General, Visceral and Vascular Surgery, University Hospital of Jena, Jena, Germany; 2 Research Programme “Clinician Scientist Programme”, Interdisciplinary Center for Clinical Research, University of Jena, Jena, Germany; 3 Department of General, Visceral and Vascular Surgery, Sophien-und Hufeland-Klinikum gGmbH, Weimar, Germany

**Keywords:** Adenocarcinoma, Squamous cell carcinoma, Anal region, Abscess, Fistula

## Abstract

**Purpose:**

Abscess or fistula of the anal region is an uncommon presentation of malignancy. Under the assumption of a benign condition, diagnostics is often delayed, resulting in advanced tumour stages at first diagnosis. Due to the case rarity, treatment guidelines for cancers of anorectal region masquerading as abscess or fistula are missing.

**Methods:**

We analysed all patients presenting with an abscess or fistula of the anal region in our department between January 2004 and August 2020. The malignancies were included to our study to acquire data on clinical presentation, treatment and outcome. Furthermore, a systematic review to present adenocarcinomas and squamous cell carcinomas associated to an abscess or fistula was performed.

**Results:**

0.5% of the patients treated for an abscess or fistula of the anal region met the selection criteria. Mean time from the onset of symptoms to diagnosis of malignancy was 100 days. Histology revealed adenocarcinoma and squamous cell carcinoma each in two patients. All patients had locally advanced tumours without distant metastases, in two cases with regional lymph-node metastases. Neoadjuvant chemoradiation was applied in two patients. All patients underwent abdomino-perineal resection of the rectum. The overall outcome reveals a recurrence-free survival of 4.5 and 3 years for two patients. Further two patients died within 5 months after the primary resection.

**Conclusion:**

Advanced carcinomas of the anorectal region may masquerade as abscess or fistula, cause diagnostic problems and delay oncologic treatment. However, even in these very advanced situations, surgical therapy with curative intent should be attempted.

## Introduction

Infections of the anal, perianal, perineal, sacral or gluteal region, such as abscesses and fistulas, are a common occurrence in surgical practice. The incidence of perianal abscesses is estimated around 15–20 per 100,000 inhabitants (Adamo et al. 2016). Sporadic fistulas occur in approximately one-third of the patients with perianal abscesses; furthermore, they are observed in 5–40% of patients with Crohn’s disease (Gold et al. 2018).

In contrast, malignant tumours of the same region are rare and represent approximately 1.5% of gastrointestinal malignancies (Salati and Al Kadi 2012). However, malignant tumours of the anal region can masquerade as inflammatory changes of the skin or fistulas, often rendering timely diagnosis (D_S_) extremely difficult (Klas et al. 1999; Leong et al. 2019; Ohta et al. 2013). Therefore, these patients usually present with advanced tumour stages (Ohta et al. 2013; Kapiteijn et al. 2001). For these rare cases, there is no consensus regarding diagnostic and treatment strategies (Yang et al. 2009). Previous reports mostly consist of case reports, in which multimodal treatment and extensive surgery are recommended, despite differences in the histological findings (Leong et al. 2019; Benjelloun et al. 2012; Gaertner et al. 2008; Pai et al. 2015).

Perianal malignancies arising from abscesses or fistulas can be adenocarcinomas (ADC) or squamous cell carcinomas (SCC). Of these two entities, adenocarcinomas are more common (2.9–10%) (Chandramohan et al. 2010; Marti et al. 2001). The adenocarcinomas can be divided into 3–6 further subgroups, of which mucinous adenocarcinomas (MAC) are reported most frequently in the context of fistulas and abscesses (Yang et al. 2009; Marti et al. 2001; Diaz-Vico et al. 2019; Maternini et al. 2018; Venclauskas et al. 2009). Mucinous adenocarcinomas derive from epithelial tissue and according to WHO classification, the diagnosis requires the secretion of extracellular mucin more than 50% of the tumour volume (Xie et al. 2018; Bosman 2010). The impact of mucinous histology on prognosis in colorectal adenocarcinomas however is yet unclear, with some studies revealing shorter overall survival compared to non-MACs (Xie et al. 2018; Soliman et al. 2016) and others, who did not find any adverse prognostic effect (Xie et al. 2018; Hogan 2013). A population-based study using the data from the Surveillance, Epidemiology and End Results (SEER) program reported a difference in survival outcomes of mucinous adenocarcinomas depending on primary tumour site; therefore, tailored treatment should be applied (Xie et al. 2018).

The aim of this paper is to report our experiences with patients who were treated for an abscess or fistula of the perianal skin and finally diagnosed with cancer. Clinical presentation, multidisciplinary management, surgical procedures and outcome are presented. Furthermore, we performed a systematic review to compare our data with previous reports, focusing on non-MACs and SCCs diagnosed in abscesses or fistulas in the perianal region.

## Methods

### Retrospective data analysis

All patients who presented in our institution between January 2004 and August 2020 with an abscess or fistula of the anal, perianal, perineal, sacral or gluteal region and eventually underwent surgery for a malignant tumour were included in this survey. The data were extracted from our electronic medical records (SAP^®^ i.s.h.med^®^) using following ICD-10 codes: K61.0–K61.4, L02.3, K60.0–K60.5. External patient data were archived in our records for patients primarily treated in peripheral hospitals and referred to us due to the case complexity. Basic demographic data, information on clinical presentation, time to diagnosis, treatment and outcome were included in the analysis. Collection of the patient’s data and the study were approved by the local ethics committee of the University Hospital Jena under the registration number 2020–2001. Mean was used for continuous variables. Statistical analysis was performed with SPSS software (IBM Company, version 23, IBM Corporation, Armonk, NY, USA).

### Systematic review

A systematic search was performed using the database Medline^®^ Library. The search was last actualized in September 2020. Inclusion criteria were full paper publications in peer-reviewed journals reporting original works, case reports or systematic reviews.

The search string for ADC was “adenocarcinoma”[Title] AND “abscess”[Title], “adenocarcinoma”[Title] AND “fistula”[Title] and “adenocarcinoma”[Title] AND “chronic infection”[Title]. For SCC, a similar string was used (“squamous cell carcinoma”[Title] AND “abscess”[Title], “squamous cell carcinoma”[Title] AND “fistula”[Title] and “squamous cell carcinoma”[Title] AND “chronic infection”[Title]). A filter was set for language (English), and mucinous adenocarcinomas, hidradenitis suppurativa as well as chronic inflammatory diseases such as Crohn’s disease were excluded.

**Fig. 1:**
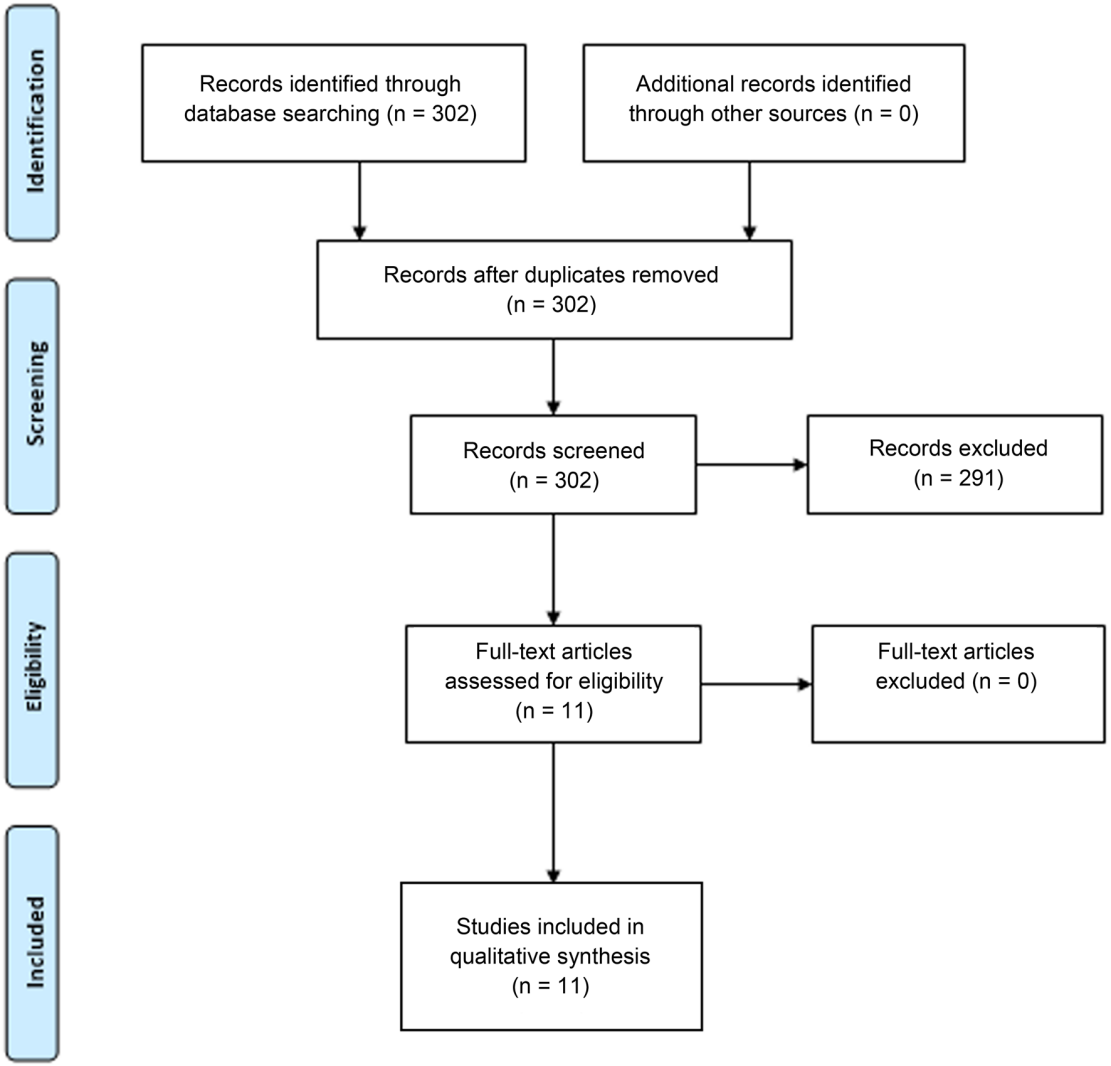
Assessment of eligible articles according to PRISMA 2009 Flow Diagram (Moher et al. 2009)

According to the title/abstract screening, all publications meeting the inclusion criteria were retrieved as full text. The data extracted included the study authors, the publication year, the number of cases, the treatment, and the outcome. The review was performed in accordance with the PRISMA statement (Fig. 1, Moher et al. 2009).

## Results

### Retrospective data analysis (Table 1)

**Table 1:** Retrospective analyses: demographic and clinical data of 4 patients of our department with delayed diagnosis of malignancy after abscess treatment

ID	Age at t of D_S_ and sex	Age at DOD	Clinical presentation at 1st consultation	t from onset of sx till 1st consultation (d)	Treatment at 1st consultation	t from onset of sx to *D*_S_ of malignancy (d)	Neoadjuvant therapy	Oncologic operation after *D*_S_ of malignancy
c1	53, m	54	10 kg weight loss in 3 mths Aanal pain Secretion of blood and pus	61	Surgical debridement Lavage	97	CR (5-FU plus Mitomycin C; 45 Gy)	APR Extensive resection of skin and gluteus muscle Resection of S3-5 Reconstruction with free latissimus muscle flap
c2	42, m	/	8 kg weight loss in 3 mths Foul-smelling secretion Fatigue Sacral wound (14 × 12 × 6 cm)	120	APR Extensive resection of skin and gluteus muscle Resection of S3-5 AV-loop Reconstruction with free latissimus muscle flap	124	None	Performed after 1st consultation
c3	53, f	/	Gluteal pain and swelling	4	Surgical debridement Necrosectomy Antibiotics	172	CR (5-FU; 45 Gy)	Posterior pelvic exenteration Resection of S3-5 Reconstruction with gluteus muscle flap
c4	70, f	71	Gluteal pain and swelling	7	Wound debridement Biopsy rectum Sigmoidostoma	8	None	APR Reconstruction with VRAM-flap

ID	Tumour marker	Histology	Clavien–Dindo	LOS after oncologic operation (d)	Adjuvant therapy	Outcome	DRFS/LRFS (mths)
c1	Ca 19–9 13.9 U/ml CEA 3.9 ng/ml	SCC T4N3M0, R0	V	101	None	Death on POD 101	/
c2	Ca 19–9 < 13 U/ml CEA 3.8 ng/ml	SCC T3N0M0, R0	IIIb	70	None	No evidence of disease	54
c3	CA 19–9 556.7 U/ml	ADC T3N0M0, R0	IIIb	49	None	No evidence of disease	36
c4	CA 19–9 < 13 U/ml CEA 3.7 ng/ml	ADC T4N1bM0, R0	I	16	CR (5-FU; 50,4 Gy)	Death on POD 178	2

*m* male, *f* female, *t* time, *sx* symptoms, *D*_*S*_ diagnosis, *DOD* date of death, *mths* months, *LOS* length of hospital stay, *AV-loop* arteriovenous loop, *VRAM-flap* vertical rectus abdominis myocutaneous flap, *POD* postoperative day,* CR* chemoradiation, *APR* abdomino-perineal resection, *DRFS* distant recurrence-free survival, *LRFS* local recurrence-free survival, *d* days

#### Demographic data

Between January 2004 and August 2020, 807 patients have been treated for anal, perianal, perineal, sacral or gluteal abscess or fistula in our department. The majority of these patients were male (m) (72%). Four patients of this cohort with an abscess of the gluteal region were later diagnosed with a malignancy. The clinical and demographical features are depicted in Table 1. The mean age at the time of tumour diagnosis was 54.5 years (y); two patients were male and two female (f).

#### Clinical presentation and time from symptoms onset to therapy (Fig. 2)

**Fig. 2:**
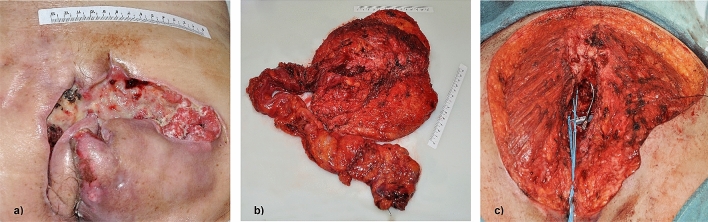
Exemplary presentation of case 1 (Table 1). **a** Clinical presentation at time of consultation in our department, **b** histological specimen after APR with extensive resection of skin, gluteus muscle and S3-5, **c** wound surface after resection prior to free latissimus muscle flap

All patients primarily presented in external hospitals after a mean time of 48 days following the onset of the symptoms. Three of the four patients reported gluteal or anal pain as the leading symptom; the remaining patient sought medical advice for a non-healing wound at the sacrum. Consequently, the primary treatment at the external hospitals consisted of drainage/excision of the presumed abscess in two of the four patients. The other two patients were referred to our department shortly after clinical examination under the suspicion of a malignant tumour (1 and 18 days after the first medical consultation). The mean time of referral to our department following the first medical consultation for all patients was 55 days. This results in a mean time from the onset of symptoms to first diagnosis of malignancy as much as 100 days. With two patients having received neoadjuvant chemoradiation, the mean time from the first symptoms to the first oncological surgery was 167 days.

#### Preoperative staging

**Fig. 3:**
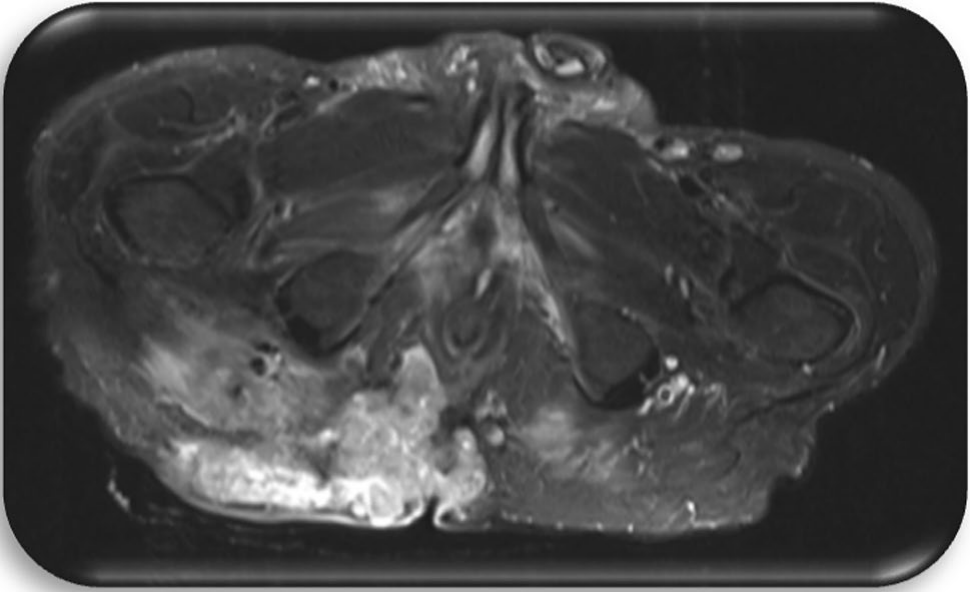
Patient c1; first MRI of the pelvis, 8 days prior to first histology of malignancy: Signal enhancement around the femoral head on the right as well as the right sacrum. Pathologically enlarged lymph nodes bilaterally in the groin area. Fistulas in the subcutaneous tissue. Inflammation in the gluteal muscles right > left. Fistula-like fluid accumulations along the inflammatory areas, minor fluid accumulations presacral and dorsal to the rectum

**Fig. 4:**
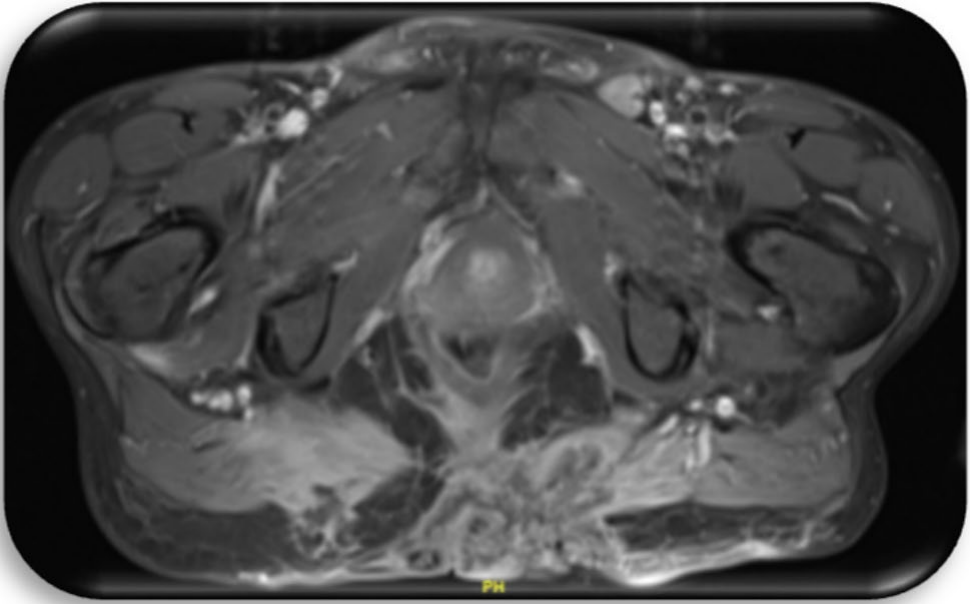
Patient c2; first MRI of the pelvis, 1 day prior to first histology of malignancy: Large, ulcerated, space occupying lesion median/paramedian on both sides gluteally from sacral vertebrae 3 to the pelvic floor, approximately 14 cm × 12 cm × 6 cm in size. Irregular configuration at the margins. Extension of the lesion cutaneously, subcutaneously and muscularly into the adjacent parts of the gluteus maximus, minimus and medius muscles as well as the piriformis muscle and the levator ani muscle. Further extension to the sacrum and the coccyx, which appears destructed. Perifocal edema. Lymph node with contrast medium enrichment at left gluteus. Pathologically enlarged iliac and inguinal lymph nodes bilaterally

**Fig. 5:**
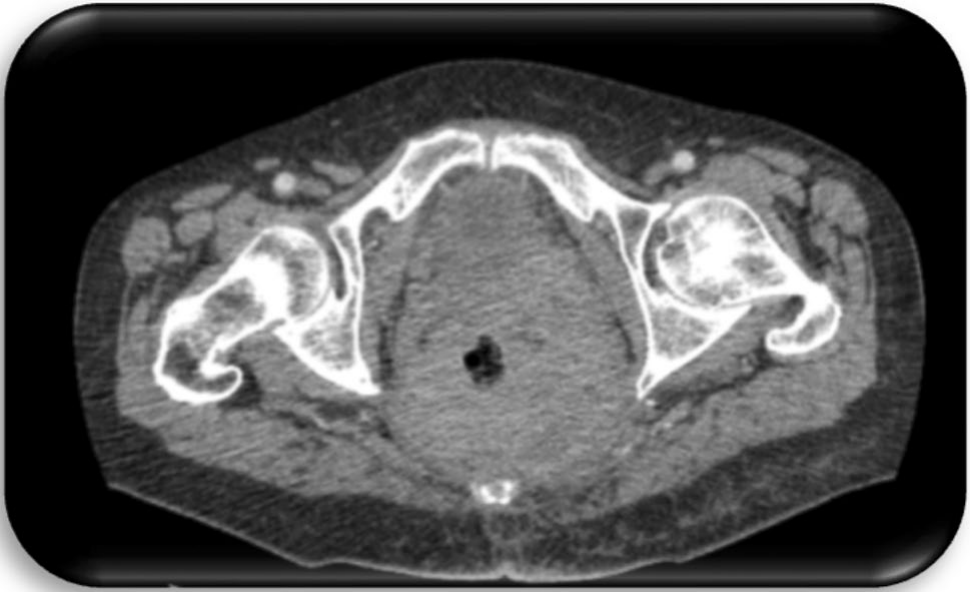
Patient c3; first CT of the pelvis, 1 day prior to first histology of malignancy: Space occupying lesion in the small pelvis with right shift of the bladder and affection of the sigmoid

**Fig. 6:**
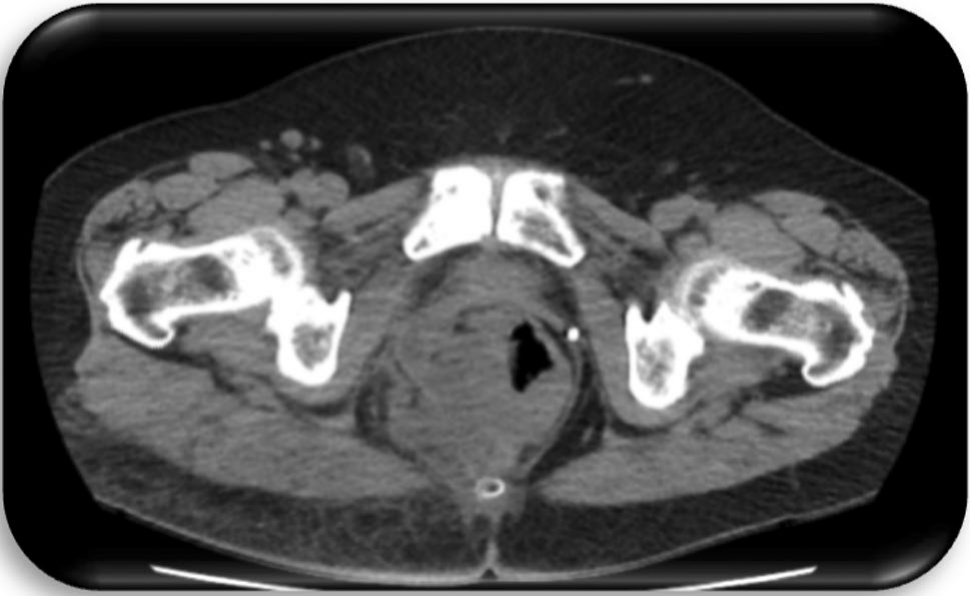
Patient c4; first CT of the pelvis, 1 day prior to first histology of malignancy: Suspicion of a large, abscess forming inflammatory lesion pararectally with air entrapments and therefore suspicious of a connection to the rectum. No evidence of fistula. Diffuse inflammatory swelling of the gluteal muscles and the subcutaneous tissue at right gluteus. Pathologically enlarged lymph nodes in the ischiorectal fossa and presacral

Preoperative diagnostics included a computer tomography (CT) of the chest, the abdomen and the pelvis, a magnetic resonance imaging (MRI) of the pelvis as well as a rectoscopy/colonoscopy in all patients (Figs. 3, 4, 5, 6).

#### Surgical treatment

All patients underwent abdomino-perineal resection of the rectum including partial resection of the sacrum in three patients. For both patients with SCC (c1 and c2), extensive resection of gluteus maximus muscles and skin was required to achieve free resection margins. The Nigro protocol could not be applied, because the clinical findings were consistent with squamous cell carcinoma of the perianal skin with deep infiltration rather than classic anal carcinoma. Furthermore, taking into account the extension of the tumour, the irradiation field would have been too large. Secondary reconstruction was performed using free latissimus-dorsi muscle flaps. In a third patient (c4), simultaneous reconstruction could be achieved with a vertical rectus abdominis myocutaneous flap (VRAM-flap).

#### Histological findings

Histology revealed ADC in both female patients and SCC in the two male patients. All patients had locally advanced tumours (pT 3 or 4) without distant metastases at the time of diagnosis, in two cases with regional lymph-node metastases (pN3 and pN1b).

#### Outcome

The mean length of hospital stay (LOS) was 59 days due to a prolonged wound healing necessitating recurrent operative wound debridements in two patients (c2 and 3, Clavien–Dindo IIIb).

One male patient with associated hemophilia A died 3 months after the tumour operation due to necrosis of the latissimus flap requiring multiple operative interventions (c1). Furthermore, a pleural carcinosis leading to respiratory failure was diagnosed in the postoperative course. Distant metastases (metachronous liver metastases) occurred in a patient 2 months after the resection of the primary tumour; she died due to liver and respiratory failure caused by an influenza infection 28 days following the partial liver resection (c4). The remaining two patients are tumour-free after a follow-up of 54 and 36 months after the oncological operation.

### Systematic review

The search disclosed a total of 302 articles containing the keywords ADC or SCC and abscess or fistula (Fig. 1). 11 studies covering the period from 1982 to 2019 met our selection criteria; 4 of them reporting ADC and 7 SCC.

## Discussion

Initially, 302 records containing our previously mentioned keywords were found with database searching. 291 articles were excluded after abstract screening for not matching the inclusion criteria. The most frequent histology found in the tumours were MACs (Leong et al. 2019; Gaertner et al. 2008; Gomes et al. 2014; Rakoto-Ratsimba et al. 2006). Due to their largely unknown histopathological behaviour (Xie et al. 2018) and to enable a comparability with our patients, MACs were excluded from analyses.

### Adenocarcinoma associated with abscess or fistula, *n* = 4 (Table 2)

Except Benjelloun et al. (2012), all authors presenting ADC associated with abscess or fistula, reported both MACs and non-MACs. However, as mentioned, only the cases with non-MACs were included to our analyses. The patients were all male and had a history of recurrent perianal infection (0.5–40 years) with the majority suffering from fistula.

**Table 2:** Systematic review: ADC associated with abscess, fistula and chronic inflammation

R	Author, Year	Article type	*n* cases	Age at t of D_S_ and sex	Clinical presentation at 1st consultation	Duration of recurrent sx	Treatment at 1st consultation
Tan et al. (1989)	Tan et al., 1989*	Case report	2	76, month	2 fistulas, induration	30 year	Sigmoid colectomy, excision of upper rectum, fistulectomy; 2^nd^ step: wide perineal resection with excision of the anorectal stump Reconstruction with inferior gluteal thigh flap
Benjelloun et al. (2012)	Benjelloun et al., 2012	Case report, literature review	2	c1: 55, month c2: 68, month	c1: anal fistula, external openings bilaterally in the perianal region, internal opening posteriorly c2: perianal abscess with internal opening in anal dentate line	c1: 10 years c2: not declared	c1: fistulectomy c2: surgical debridement
Hongo et al. (2013)	Hongo et al., 2013**	Case report, literature review (original paper)	11	c1: 69, month c2: 74, month c3: 74, month c4: 54, month	c1: secretion, fistula c2: pain, fistula c3: pain, fistula c4: pain, mass, fistula	c1: 3 years c2: 0.5 years c3: 40 years c4: 30 years	c1: none c2: none c3: fistulectomy c4: fistulectomy, multiple drainages
Leong et al. (2019)	Leong et al., 2019***	Case report	5	72, months	Perianal secretion, pain, bleeding, lump at anus	5 years	Not declared

R	t from 1st clinical presentation to *D*_S_ of malignancy	Neoadjuvant therapy	Oncologic operation after D_S_ of malignancy	Histology	Adjuvant therapy	Outcome	DRFS/LRFS
Tan et al. (1989)	At 1st consultation	None	Performed after 1st consultation	ADC, T3N0, R0	None	Not reported	Not reported
Benjelloun et al. (2012)	c1: 3 months c2: at 1st consultation	c1: CR (5-FU; 45 Gy) c2: CR (5-FU; 45 Gy)	c1: APR, local excision of perianal mass c2: APR, local excision of perianal mass	c1: ADC, CK20 + , CK 7-; T3N0M0 c2: ADC, CK20 + , CK7 + ; T2N0M0	None	c1: no evidence of disease c2: no evidence of disease	c1: 3 years c2: 3 years
Hongo et al. (2013)	c1–c4: at 1st consultation	c1–c4: none	c1–c4: APR	c1: ADC, T2N0M0 c2: ADC, T3N2M0 c3: ADC, T2N0M0 c4: ADC, T3N0M0	c1–c3: none c4: chemo-therapy	c1: no evidence of disease c2: death due to other disease c3: no evidence of disease c4: death due to local recurrence after 104 months	c1: 11 months c2: 19 months till death c3: 52 months c4: unclear
Leong et al. (2019)	At 1st consultation	CR	No surgery (metastases)	ADC, T4N0M0	None	death due to metastases upon completion of CR	/

*R* reference, *n* number, *CK* cytokeratin, *RT* radiotherapy

*Out of 2 reported cases, 1 was non-mucinous ADC. The remaining case was not reported

**4/11 cases with non-mucinous ADC

***1/5 cases with non-mucinous ADC

The first case described by Benjelloun et al. (2012) had a history of anal fistula of 10 years; 3 months after the last fistulectomy, an ADC was diagnosed. In all remaining case reports, the tumour diagnosis was at first clinical treatment (Leong et al. 2019; Benjelloun et al. 2012; Hongo et al. 2013; Tan et al. 1989).

Tan et al. (1989) performed a barium enema study that showed an irregular narrowed area of the sigmoid colon; further staging modalities are not reported. Benjelloun et al. (2012) and Leong et al. (2019) staged the patients with preoperative CT scan, MRI and endoscopy. Hongo et al. (2013) mostly performed a contrast enhanced CT scan and only added MRI in a few cases to assess the local extent of disease. Additionally, both cases reported by Benjelloun et al. (2012) received endoanal ultrasound.

The majority of the patients initially received local surgical therapies for infect control. Tan et al. (1989) immediately performed sigmoid colectomy with excision of upper rectum and fistulectomy. At the second step, a wide perineal resection with excision of the anorectal stump was done to have tumour-free margins. An inferior gluteal thigh flap was used for reconstruction of the defect. The patient reported by Leong et al. (2019) was treated with chemoradiation due to metastatic disease. All other patients received APR with (Benjelloun et al. 2012) or without (Hongo et al. 2013) local excision of perianal mass.

The outcome of the patients described in the cited cases varies. Tan et al. (1989) did not indicate the outcome. The patient reported by Leong et al. ( 2019) died due to the development of metastases 5 months after first diagnosis upon completion of CR for a pT4 N0 M0 ADC. Distant recurrence-free survival and local recurrence-free survival ranged from 11 to 52 months in the cases presented by Benjelloun et al. (2012) and Hongo et al. (2013). The maximum survival was 104 months (c4, Hongo et al. 2013) with death due to local recurrence. However, the exact time of recurrence is unclear.

### Squamous cell carcinoma associated with abscess or fistula, *n* = 7 (Table 3)

**Table 3:** Systematic review: SCC associated with abscess, fistula and chronic inflammation

R	Author, year	Article type	*n* cases	Age at t of *D*_S_ and sex	Clinical presentation at 1st consultation	Duration of recurrent sx	Treatment at 1st consultation
Jamieson and Goode (1982)	Jamieson et al., 1982	Case report	1	63, unclear	3 month history of swelling around pilonidal sinus with 3 openings, slowly increasing in size Persistent purulent secretion from the sinus No ulceration No inguinal lymph-adenopathy	20 years	Complete excision of sinus and abscess
Seya et al. (2007)	Seya et al., 2007	Case report	1	57, f	Anal pain since 6 months 3 external fistula openings, 1 internal Induration anal region revealing anal inter-sphincteric fistula	32 years	Fistulectomy
Chandramohan et al. (2010)	Chandramohan et al., 2010	Case report	1	56, m	Recurrent abscess of 4 month duration at right gluteal area in preexisting perianal fistulae Ulceration Purulent secretion	32 years	Local excision with wide margins Reconstruction with gluteal rotation flap
Moore et al. (2016)	Moore et al., 2016	Case report	1	Late 40 s, m	1st: perineal abscess 2nd: 1 year later scrotal edema and abscess with urethra-cutaneous fistula, purulent and necrotic tissue 3rd: further 6 months later Fournier’s gangrene	32 years	1st: surgical debridement 2nd: antibiotics and VAC therapy 3rd: drainage and further debridement of necrotic tissue
Creta et al. (2017)	Creta et al., 2017	Case report	1	78, m	1st: perineal pain, purulent discharge, acute urinary retention 2nd: 6 months later perineal pain, bleeding from perineal wound	No past history	1st: urinary catheter, drainage of abscess, excision of suspect urethra-cutaneous fistula
Garg et al. (2018)	Garg et al., 2018	Case report	1	65, m	1st: urinary tract syndromes and perineoscrotal swelling 2nd: 3 months later non-healing wound perineum and urine passage from wound; urethra-cutaneous fistula	No past history	1st: incision, drainage, suprapubic catheter
Mizusawa et al. (2019)	Mizusawa et al., 2019	Case report	1	69, m	1st: pain on urination 2nd: swelling of scrotum and perineum, purulent secretion, partially necrotized scrotal skin 3rd: after 3 weeks with urinary incontinence from perineal wound, purulent secretion 4th: remaining abscess in MRI	No past history	1st: antibiotics 2nd: incision, wound debridement, antibiotics 3rd: percutaneous cystostomy, wound opening, drainage, antibiotics 4th: resection of infected tissue in perineal region

R	t from 1st clinical presentation to D_S_ of malignancy	Neoadjuvant therapy	Oncologic operation after D_S_ of malignancy	Histology	Adjuvant therapy	Outcome	DRFS/LRFS
Jamieson and Goode (1982)	At 1st consultation	None	Hemi-corporectomy after 3rd recurrence denied	SCC	RT (after 2nd recurrence)	Several local recurrences (1st after 3 months, 2nd/3rd after further 3 months/6 months); each with local excision Death 18 months after 1st D_S_	3 months
Seya et al. (2007)	At 1st consultation	None	APR with lymph-node dissection	SCC	None	8 years after D_S_ of SCC, D_S_ of urothelial carcinoma in the bladder Death 2 years after resection of urothelial carcinoma due to disseminated disease	10 years
Chandramohan et al. (2010)	At 1^st^ consultation	None	/	SCC	None	Not reported	Not reported
Moore et al. (2016)	18 months	None	Penectomy, urethrectomy, scrotectomy, bilateral orchidectomy, bilateral pelvic lymph-adenectomy and groin dissection, resection of inferior pubic rami with urogenital diaphragm, rectum resection with permanent end colostomy Reconstruction via anterolateral thigh skin flap closure	SCC of urethra, T4 N2M1	CR 3 months post-surgery, chemo-therapy 6 months post-surgery	Death 2 years after 1st consultation due to metastatic disease	3 months
Creta et al. (2017)	6 months	None	No tumour resection due to patients’ bad general condition, only colostomy	1st: chronic inflammation 2nd: SCC in pelvic tumour, CUP	None	Rapid worsening of general condition	Lost to follow up after 3 months
Garg et al. (2018)	3 months	CR	Patient refused en bloc resection	SCC of urethra	None	Not reported	Not reported
Mizusawa et al. (2019)	2 months	None	En bloc urethral tumour resection and lymph-node dissection	SCC of urethra	RT 45 Gy, chemo-therapy	Gradual worsening due to metastatic disease and local recurrence Death 17 months after surgery	Time of recurrence not reported

*VAC* vacuum-assisted closure, *CUP* carcinoma of unknown primary origin

The authors reported each 1 case of SCC associated with abscess or fistula (Chandramohan et al. 2010; Jamieson and Goode 1982; Seya et al. 2007; Moore et al. 2016; Creta et al. 2017; Garg et al. 2018; Mizusawa et al. 2019). In the majority of the cases, the patients were male (5/7), only 1 was female, and in 1 case, the sex was not reported.

4/7 patients had a history of recurrent infection for more than 20 years prior to the diagnosis of malignancy. The infections, either acute or chronic, have been in the anal, perineal or gluteal area. The patient presented by Jamieson et al. (1982) had a 20 years history of pilonidal sinus and the female patient reported by Seya et al. (2007) suffered from recurrent perianal abscesses with fistulas since 32 years. In one patient, the abscess was located in the gluteal region (Chandramohan et al. 2010). In the remaining 4 case reports, the infection was found in the perineal region followed by the delayed diagnosis of a urethral tumour (3 cases) (Moore et al. 2016; Garg et al. 2018; Mizusawa et al. 2019) or a carcinoma of unknown primary origin (CUP) (Creta et al. 2017) (delay of 2–18 months).

The staging examinations were not performed in a standardized way. For instance, Jamieson et al. (1982) performed an isotopic bone and a CT scan only after the third recurrence of the tumour; Seya et al. (2007), Chandramohan et al. (2010) and Creta et al. (2017) carried out endoscopic examination prior to the operation. Furthermore, Chandramohan et al. (2010), Moore et al. (2016), Creta et al. (2017), Garg et al. (2018) and Mizusawa et al. (2019) completed the staging with a cross-sectional imaging.

All patients were treated with minor local surgery at first presentation. In the case described by Jamieson et al. (1982), the first two recurrences were also treated by local resection. However, after the third recurrence, hemi-corporectomy was offered which was refused by the patient. For the patient reported by Seya et al. (2007), an APR with lymph-node dissection was performed. No further surgery was done for the patient in Chandramohan et al.s (2010) case report. Due to the delayed diagnosis of malignancy in the patients with urethral tumour and CUP, an extended resection was necessary (Moore et al. 2016; Creta et al. 2017; Garg et al. 2018; Mizusawa et al. 2019). However, only two patients were eligible for surgery (Moore et al. 2016; Mizusawa et al. 2019), one was in a poor general condition and therefore not suitable for en bloc tumour resection (Creta et al. 2017) and a further one refused an extended resection (Garg et al. 2018).

The outcome of all patients reported can be considered as rather poor. For two patients, the outcome was not mentioned (Chandramohan et al. 2010; Garg et al. 2018), and one patient was lost to follow up after 3 months (Creta et al. 2017). Three patients died after 17 (Mizusawa et al. 2019), 18 (Jamieson and Goode 1982) and 24 (Moore et al. 2016) months, respectively. The patient in Seya et al. (2007) report showed a recurrence-free survival of the SCC in the anal region for 10 years. However, 8 years after the first tumour diagnosis, an urothelial carcinoma of the urinary bladder was found, and the patient died 2 years after the resection of the second tumour due to disseminated disease.

The systematic review reveals that consistent treatment concepts are missing. Individual decisions are taken concerning diagnostics and therapy, according to the tumour extension. This might be due to the case rarity. In the reported cases for both entities, there was a history of recurrent abscess or fistula for 0.5–40 years. This gives rise to the assumption, that the tumour derived due to the chronic infection (malignant transformation). It remains unclear, if the tumour diagnosis in these cases was delayed, because a long history of disease with frequent recurrence (Leong et al. 2019; Benjelloun et al. 2012; Chandramohan et al. 2010; Jamieson and Goode 1982; Seya et al. 2007; Moore et al. 2016) can often lead to an underestimation of the severeness. However, it is well known that the long-term risk of anal cancer is significantly increased in patients with inflammatory anal lesions (Nordenvall et al. 2006). Since Virchow’s hypothesis in 1863, namely that lymphoreticular infiltrate reflected the origin of cancer at sites of chronic inflammation (Virchow 1863), several types of cancer have been associated with infections (Balkwill and Mantovani 2001).

The prevalence of perianal abscess in men is higher (Adamo et al. 2016). Also, the incidence rate of invasive anal carcinoma in the United States is reported to be higher in men (Benson et al. 2018). The gender distribution in our systematic review depicts the trend that the occurrence of ADC and SCC seems to be more common in male patients. In our retrospective study, 2 of the tumour patients were male and 2 female.

In general, the malignancy appears to arise at a later age: the mean age at the time of diagnosis in the literature reviewed was 64.7 years (exact age was missing in one patient) and 54.5 years in our data set.

The case numbers in our department (*n* = 807 in 16 years) confirm the relatively high incidence of abscesses or fistulas in the anal region (Adamo et al. 2016) requiring minor surgical interventions. Furthermore, with only 0.5% of malignancy within our data set, our experience confirms that this condition is rather rare (Salati and Al Kadi 2012; Kline et al. 1964).

However, following the analysis of our data set, we report an important new finding. Malignancies can be associated with abscesses of the anal, perianal, perineal, sacral or gluteal region even if the history of disease is rather short compared to those described in the literature. The lesions often appear benign, and therefore, histological examination is frequently not performed at the time of initial diagnosis of an abscess (Moore et al. 2016; Garg et al. 2018), or false-negative biopsies occur (Leong et al. 2019). This considerably impedes the timely diagnosis, leads to advanced tumour stages and renders the therapy extremely difficult.

We can assume that both the patient and the surgeon are involved in the cause of late diagnosis. In our cohort, the patients presented after 48 days following the onset of their symptoms. After approximately twice the time, 100 days, the malignancy was proven. Due to a missing consensus for the treatment of those patients, individual therapy concepts have to be designed (Yang et al. 2009).

All of our patients received an extensive oncological surgery. 2 of them (c1 and c3) had neoadjuvant therapy, 1 (c4) adjuvant therapy and 1 (c2) had neither neoadjuvant nor adjuvant therapy. With this treatment concept, 2 of our 4 patients present a long-term survival with DRFS/LRFS of 4.5 (c2) and 3 (c3) years. Compared to the data in the literature review, this is a favourable outcome.

The significance of neoadjuvant or adjuvant chemoradiation is yet unclear. In case of ADCs, it seems reasonable to adhere to the guidelines for colorectal carcinomas and to apply neoadjuvant chemoradiation in the generally advanced carcinomas. For SCC, however, the situation is less clear. Both our patients reported here had very extensive involvement of the gluteal and sacral skin, unlike anal carcinoma. Even with partial response, the extent of surgery is likely not to be reduced, which was our reason to withhold neoadjuvant treatment in the second patient with SCC.

## Conclusion

Despite the rarity of malignancies associated with abscesses and fistulas of the anal, perianal, perineal, sacral or gluteal region, histologic sample biopsies from the wound ground and/or the fistula canal should be assessed on a routine manner to prevent late diagnosis of malignancy. Especially, in cases of non-healing wounds and persistent pain, medical professionals should be suspicious. We would like to point out that, even in advanced tumour stages and regardless of the tumour entity, multimodal treatment concepts with an extensive surgery may lead to a promising outcome.

Ideally, a consensus guideline should be established for these cases to standardize treatment options and improve survival. However, due to the heterogeneity of the disease and its infrequent occurrence, this is not realistic. Treatment plans therefore should be discussed in multidisciplinary tumour boards and especially take the local resectability/need for downsizing into consideration.

## Data Availability

The datasets analysed during the current study are available from the corresponding author on reasonable request.
